# Stratification of Prognosis in Pulmonary Pleomorphic Carcinoma Based on Integrated PD-L1 and Multiparametric Biomarker Analysis

**DOI:** 10.3390/cancers18142240

**Published:** 2026-07-13

**Authors:** Yohei Honda, Shohei Shimajiri, Takehiko Manabe, Yukiko Nemoto, Rintaro Oyama, Natsumasa Nishizawa, Hiroki Matsumiya, Yusuke Nabe, Masaru Takenaka, Koji Kuroda, Fumihiro Tanaka, Hidetaka Uramoto

**Affiliations:** 1 Second Department of Surgery, University of Occupational and Environmental Health, 1-1 Iseigaoka, Yahatanishi-ku, Kitakyushu 807-8555, Fukuoka, Japan; 2 Second Department of Pathology, University of Occupational and Environmental Health, 1-1 Iseigaoka, Yahatanishi-ku, Kitakyushu 807-8555, Fukuoka, Japan

**Keywords:** biomarker combination, CD73, CD155, immunohistochemistry, Ki-67, PD-L1, prognosis, pulmonary pleomorphic carcinoma

## Abstract

Background/Objectives: Published data on reliable prognostic biomarkers for pulmonary pleomorphic carcinoma (PPC), a rare, aggressive subtype of non-small cell lung cancer, are limited. Programmed death-ligand 1 (PD-L1) has been studied; however, the prognostic impact of other immunoregulatory molecules, such as CD73, CD155, and Ki-67 and their combined expression profiles, remains unclear. Methods: We retrospectively analyzed data of 47 patients who had undergone macroscopic complete resection of PPC between January 2000 and December 2022. Immunohistochemistry for PD-L1, CD73, CD155, and Ki-67 was performed. Cut-off values were determined by receiver operating characteristic analysis for cancer-specific death. The primary endpoint was the association between each biomarker and overall survival (OS) or cancer-specific survival (CSS). The secondary endpoint was the prognostic value of combined biomarker profiles, particularly PD-L1 with CD73, CD155, or Ki-67. Results: At a positivity threshold of 1%, the positivity rates were 70.2% for PD-L1, 89.4% for CD73, 91.5% for CD155, and 83.0% for Ki-67. There were no statistically significant differences in OS or CSS between groups with high expression and those with low expression for any single marker. Secondary endpoint analysis showed that low PD-L1 and low CD73 predicted significantly poorer OS (*p* = 0.046) and low PD-L1 and low Ki-67 predicted significantly poorer CSS (*p* = 0.039) compared with other combinations. Conclusions: Primary endpoint analyses showed no statistically significant association between any single marker and prognosis in PPC. In contrast, secondary endpoint findings indicated that concurrent low PD-L1 with either low CD73 or low Ki-67 identified subsets with significantly poorer outcomes. The present findings provide a basis for further investigation of biomarker combinations for prognostic assessment in this rare lung cancer. Because of the limited sample size, these findings are exploratory and require validation in larger independent cohorts.

## Simple Summary

Pulmonary pleomorphic carcinoma (PPC) is a rare and highly aggressive subtype of lung cancer that remains difficult to treat and is associated with poor outcomes. Although immune checkpoint inhibitors targeting PD-L1 have improved outcomes in lung cancer, identifying additional biomarkers that better predict patient prognosis remains important. In this study, we evaluated four biomarkers namely PD-L1, CD73, CD155, and Ki-67 that are measurable in tumor tissue and may help predict patient outcomes. These markers were examined in tumor samples from 47 patients who had undergone surgical resection. We found that assessing these markers in combination provided more informative prognostic value than evaluating each marker individually. In particular, tumors with concurrent low expression of PD-L1 and CD73, or PD-L1 and Ki-67, were associated with significantly poorer survival. These findings suggest that multi-biomarker assessment may improve prognostic stratification in PPC and help guide clinical management.

## 1. Introduction

Pulmonary pleomorphic carcinoma (PPC), a major subtype of pulmonary sarcomatoid carcinoma (PSC), accounts for <1% of lung malignancies. It progresses rapidly, is chemoradiotherapy resistant, and has poor outcomes, even with multimodal therapy [1,2,3,4]. Actionable driver mutations are relatively uncommon in PSC/PPC, limiting the availability of targeted therapy [5,6]. Reliable prognostic and predictive biomarkers are therefore needed. Immune checkpoint inhibitors (ICIs) targeting PD-1/PD-L1 have transformed care of patients with non-small cell lung cancer (NSCLC) [7,8,9,10]. Although PD-L1 is used to guide selection of ICIs, its association with prognosis remains controversial. We have previously shown frequent PD-L1 positivity in PPC and a trend toward better prognosis in patients with high PD-L1 whose primary tumors have been resected [11]. Assessing other immune-related axes may capture the heterogeneity of the tumor microenvironment (TME). CD73 (ecto-5′-nucleotidase) drives adenosine-mediated immunosuppression and portends poor outcomes in NSCLC. This has prompted clinical testing of anti-CD73 combinations with PD-1/PD-L1 blockade [12,13]. CD155 (poliovirus receptor) engages T cell immunoreceptor with immunoglobulin and ITIM domain TIGIT to inhibit T/NK cells, thus providing an alternative checkpoint pathway. Furthermore, combinations of anti-TIGIT strategies with PD-1/PD-L1 blockade for lung cancer have also attracted clinical interest [14,15,16]. Both CD73 and CD155 are currently attracting attention as therapeutic targets in combination with PD-1/PD-L1 blockade. We therefore included these markers in the present analysis. Our aim was to determine whether combining them with PD-L1 provides additional prognostic information in patients with PPC. We evaluated Ki-67 as a marker of tumor proliferative activity separately. PPC is known to be very aggressive biologically, consistent with its pathological features, which suggest high proliferative activity [4]. We therefore evaluated the use of Ki-67 and PD-L1 together to determine whether this combination provides more prognostic information than does either marker alone. Composite biomarker approaches (e.g., PD-L1 with tumor-infiltrating lymphocytes, tumor mutational burden, gene-expression profiles, or other checkpoints) can improve prognostic/predictive power for efficacy of ICI across many cancers [17,18,19,20,21,22,23,24]. Such combinations have rarely been evaluated in PPC. The primary objective of evaluating these markers in our cohort was to investigate their baseline prognostic significance in resected PPC, rather than their predictive value for response to immune checkpoint inhibitors. We therefore assessed PD-L1, CD73, CD155, and Ki-67 individually and in combination in patients who had undergone resection of PPC, prespecifying the primary endpoint (single markers vs. OS/CSS) and secondary endpoint (combinations vs. OS/CSS).

## 2. Materials and Methods

### 2.1. Patient Cohort

We retrospectively evaluated data of patients with PPC who had undergone macroscopic complete resection at the Second Department of Surgery, University of Occupational and Environmental Health, Japan, from January 2000 to December 2022. Patients for whom insufficient tumor cells were available for analysis for immunohistochemistry (IHC) or who had not been given written informed consent for future research use of their surgical specimens and clinical data were excluded, leaving 47 patients. The study was approved by the Institutional Review Board of the University of Occupational and Environmental Health (Approval number H26-15; Kitakyushu, Japan).

### 2.2. Immunohistochemical Staining

**Figure 1:**
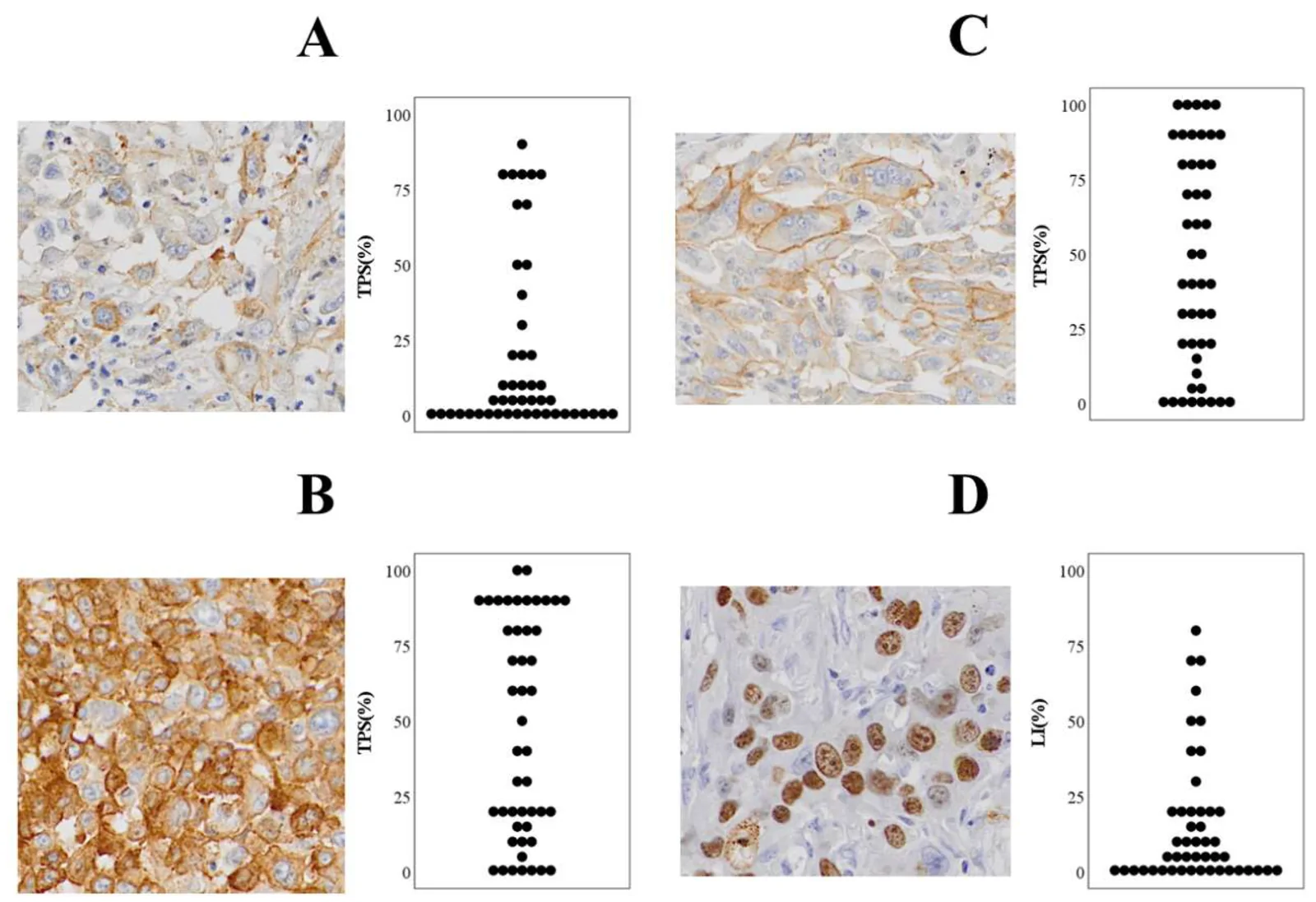
Representative immunohistochemical staining images and distribution of expression of PD-L1, CD73, CD155, and Ki-67 in pulmonary pleomorphic carcinoma. (**A**) PD-L1, (**B**) CD73, (**C**) CD155, and (**D**) Ki-67. Tumor proportion scores were used for PD-L1, CD73, and CD155, and labeling index (LI) for Ki-67. (Original magnification, ×400; Immunohistochemistry).

Sections (4 µm) from formalin-fixed paraffin-embedded blocks were stained with hematoxylin–eosin and subjected to IHC, the primary antibodies assessed being PD-L1 (clone E1L3N), CD73 (D7F9A), CD155 (D8A5G), and Ki-67 (D2H10). These were all obtained from Cell Signaling Technology (CST, Danvers, MA, USA). Our protocols were in accordance with the relevant manufacturer instructions [25]. Sections were baked at 60 °C for 30 min. Antigens were retrieved as follows: PD-L1 in 1 mmol/L EDTA (pH 8.0; 98 °C, 15 min); then CD73/CD155/Ki-67 in citrate (pH 6.0; 98 °C, 10 min), then bench cooled for 30 min. Endogenous peroxidase was then quenched (3% H_2_O_2_, 10 min). After serum-free protein blocking (Agilent, Santa Clara, CA, USA; 30 min), primaries (1:200, room temperature, 1 h) were applied, followed by SignalStain Boost (HRP-Rabbit; CST; 30 min). We used DAB+ (Agilent) slides counterstained with hematoxylin (Agilent) for detection. Two investigators (Y. H., R. O.) who were blinded to the clinical data scored the resultant slides. Inter-observer agreement for the initial high/low expression of each marker was assessed using Cohen’s kappa coefficient. Disagreements were resolved by consensus on the findings of double-headed microscopy. Membranous staining (any intensity) in tumor cells was defined as positivity for PD-L1, CD73, and CD155. Ki-67 positivity was nuclear and recorded as labeling index (%) (Figure 1).

This study extends the findings of our previous PPC series, which showed that frequent PD-L1 positivity and a 15% cut-off were associated with a favorable prognosis [11].

### 2.3. Endpoints

The primary endpoint was association between each biomarker singly (PD-L1, CD73, CD155, Ki-67) and OS/CSS. The secondary endpoint was association between combinations of biomarkers (PD-L1 with CD73, CD155, or Ki-67) and OS/CSS.

### 2.4. Statistical Analysis

**Figure 2:**
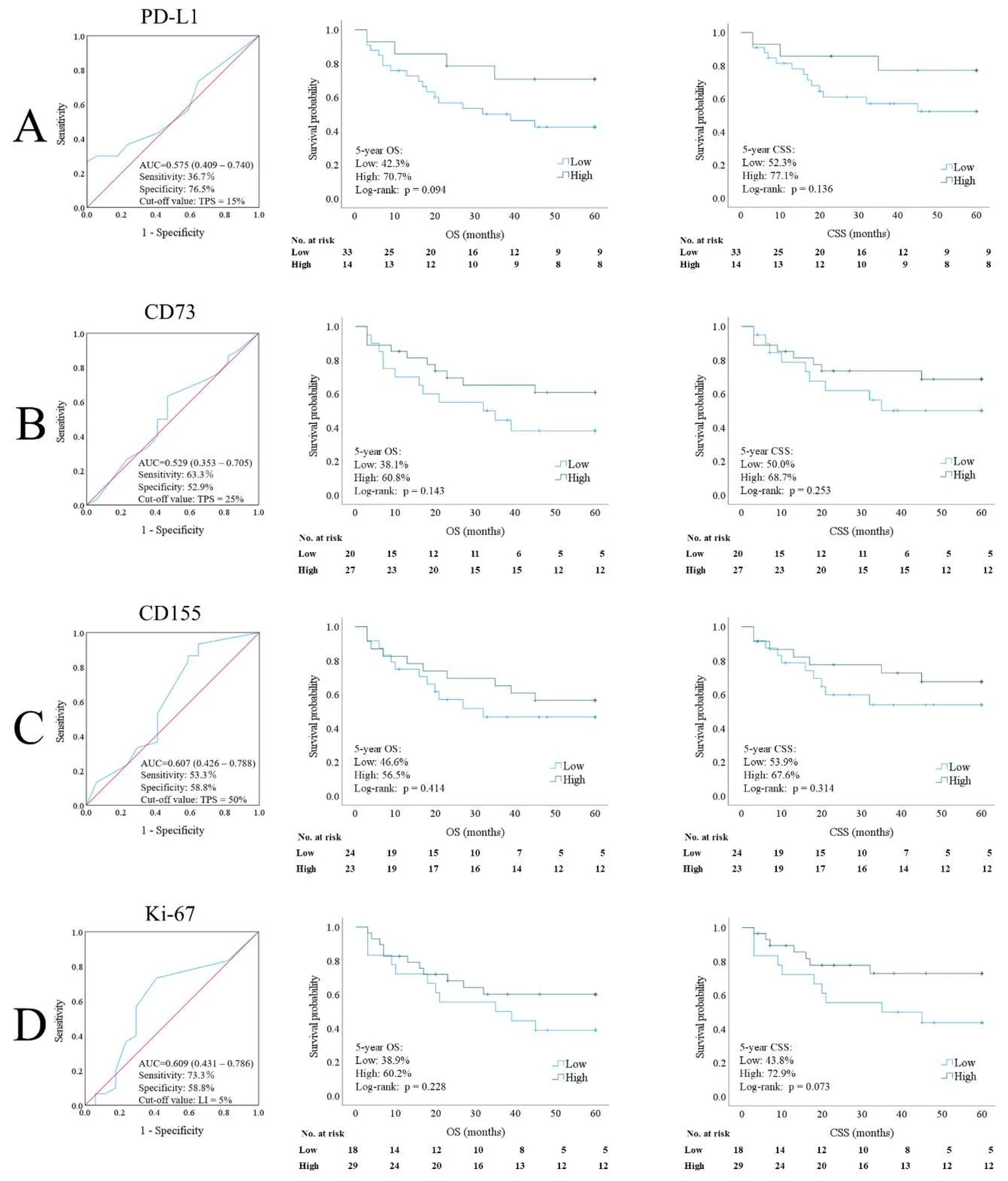
Cut-off values based on ROC curves with cancer-specific death as the outcome and Kaplan–Meier survival analyses for each molecular marker. (**A**) PD-L1, (**B**) CD73, (**C**) CD155, (**D**) Ki-67. *p*-values were determined by the log-rank test. AUC: area under the curve; ROC: receiver operating characteristic.

Because standardized cut-off values for these biomarkers have not been established in pulmonary pleomorphic carcinoma (PPC), cut-off values were determined by receiver operating characteristic (ROC) curve analysis using cancer-specific death as the outcome, in accordance with the approach used in a previous PPC study [11]. The value with the highest Youden index was selected as the optimal cut-off for each marker. The resulting cut-off values were 15% for PD-L1, 25% for CD73, 50% for CD155, and 5% for Ki-67 (Figure 2).

Overall survival (OS) and cancer-specific survival (CSS) were determined using the Kaplan–Meier method and compared using the log-rank test. For the combination analyses, PD-L1 was paired with CD73, CD155, or Ki-67. For each biomarker pair, cases were classified into four groups according to the combined high/low expression status of the two markers. Survival analyses were then performed in three ways. First, across all four groups; second, by comparing cases with concurrent high expression of both markers with all other cases; and third, by comparing cases with concurrent low expression of both markers with all other cases. Cox proportional hazards regression models were used for univariate and multivariable analyses, and hazard ratios (HRs) with 95% confidence intervals (CIs) were calculated. The variables examined by Cox regression analyses were age, sex, ECOG performance status, curative resection status, histologic subtype, pathological stage, PD-L1 expression, CD73 expression, CD155 expression, Ki-67 expression, and perioperative therapy. Two-sided *p* values < 0.05 were considered to denote statistical significance. All statistical analyses were performed using SPSS version 21 (IBM).

## 3. Results

### 3.1. Patient Characteristics

Table 1 summarizes baseline data. Men comprised 80.9% of participants. The three R1 resections all had positive chest-wall margins. Histologic examination of operative specimens revealed admixture of adenocarcinoma in 29 and squamous cell carcinoma in 3. Perioperative therapy was administered to 23/47 patients (48.9%; neoadjuvant 5 [10.6%], adjuvant 18 [38.3%]).

### 3.2. Expression of Each Immunoregulatory Molecule in Tumor Cells

Figure 1 shows representative IHC findings and the distribution of level of expression. Using 1% as the threshold for positivity, the rates of positivity were 70.2% for PD-L1, 89.4% for CD73, 91.5% for CD155, and 83.0% for Ki-67 (Table 1). The PD-L1 positivity rate was consistent with that found in our previous PPC study [11]. Initial inter-observer agreement for the immunohistochemical evaluations was good to excellent (Cohen’s κ ranged from 0.777 to 1.000; all *p* < 0.001). As described in the Materials and Methods, any initial disagreements were resolved by consensus using a double-headed microscope.

### 3.3. Prognostic Correlations and Determination of Cut-Off

ROC-derived cut-offs for cancer-specific death were as follows: PD-L1 15%, CD73 25%, CD155 50%, and Ki-67 5% (Figure 2). Patients were allocated to high (≥cut-off) versus low (<cut-off) groups.

### 3.4. Survival Analyses (Single Markers; Primary Endpoint)

Five-year OS/CSS tended to be higher in high versus low groups for all markers; however, none of these differences were statistically significant. Examples included the following: PD-L1 high versus low, OS 70.7% versus 42.3% (*p* = 0.094); CSS 77.1% versus 52.3% (*p* = 0.136); CD73 OS 60.8% (n = 25) versus 38.1% (n = 22) (*p* = 0.143); CSS 68.7% versus 50.0% (*p* = 0.253); CD155, OS 56.5% (n = 24) versus 46.6% (n = 23) (*p* = 0.414); CSS 67.6% versus 53.9% (*p* = 0.314). Ki-67 OS 60.2% (n = 29) versus 38.9% (n = 18) (*p* = 0.228); and CSS 72.9% versus 43.8% (*p* = 0.073) (Figure 2).

### 3.5. Survival Analyses (Four-Group Combinations; Secondary Endpoint)

**Figure 3:**
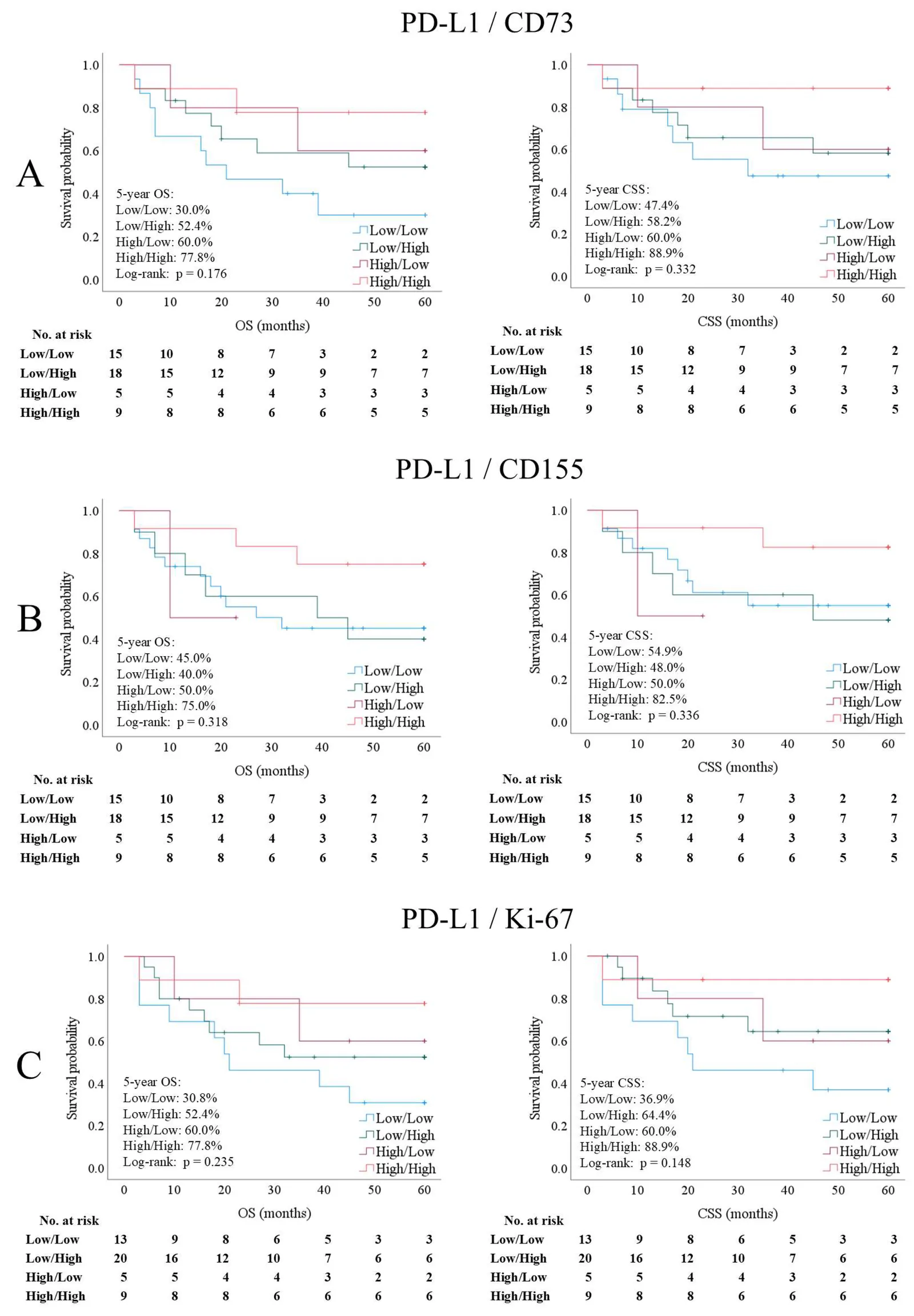
Kaplan–Meier curves for overall survival and cancer-specific survival according to the combined high/low expression status of PD-L1 and one additional biomarker. *p* values were calculated using the log-rank test. (**A**) PD-L1 and CD73. (**B**) PD-L1 and CD155. (**C**) PD-L1 and Ki-67.

For each PD-L1-based biomarker pair, cases were first classified into four groups according to the combined high/low expression status of the two markers, following which Kaplan–Meier curves were compared across these four groups (Figure 3).

No statistically significant differences in associations with OS or CSS were detected among the four groups for PD-L1/CD73, PD-L1/CD155, or PD-L1/Ki-67. However, in the PD-L1/CD73 and PD-L1/Ki-67 combinations, the low/low group tended to have the poorest survival of the four groups. Log-rank *p* values were 0.176 and 0.332 for associations with OS and CSS, respectively; for PD-L1/CD73, 0.318 and 0.336; for PD-L1/CD155, 0.235 and 0.148; for PD-L1/Ki-67 (Figure 3A–C).

### 3.6. Survival Analyses (Combinations; Secondary Endpoint)

**Figure 4:**
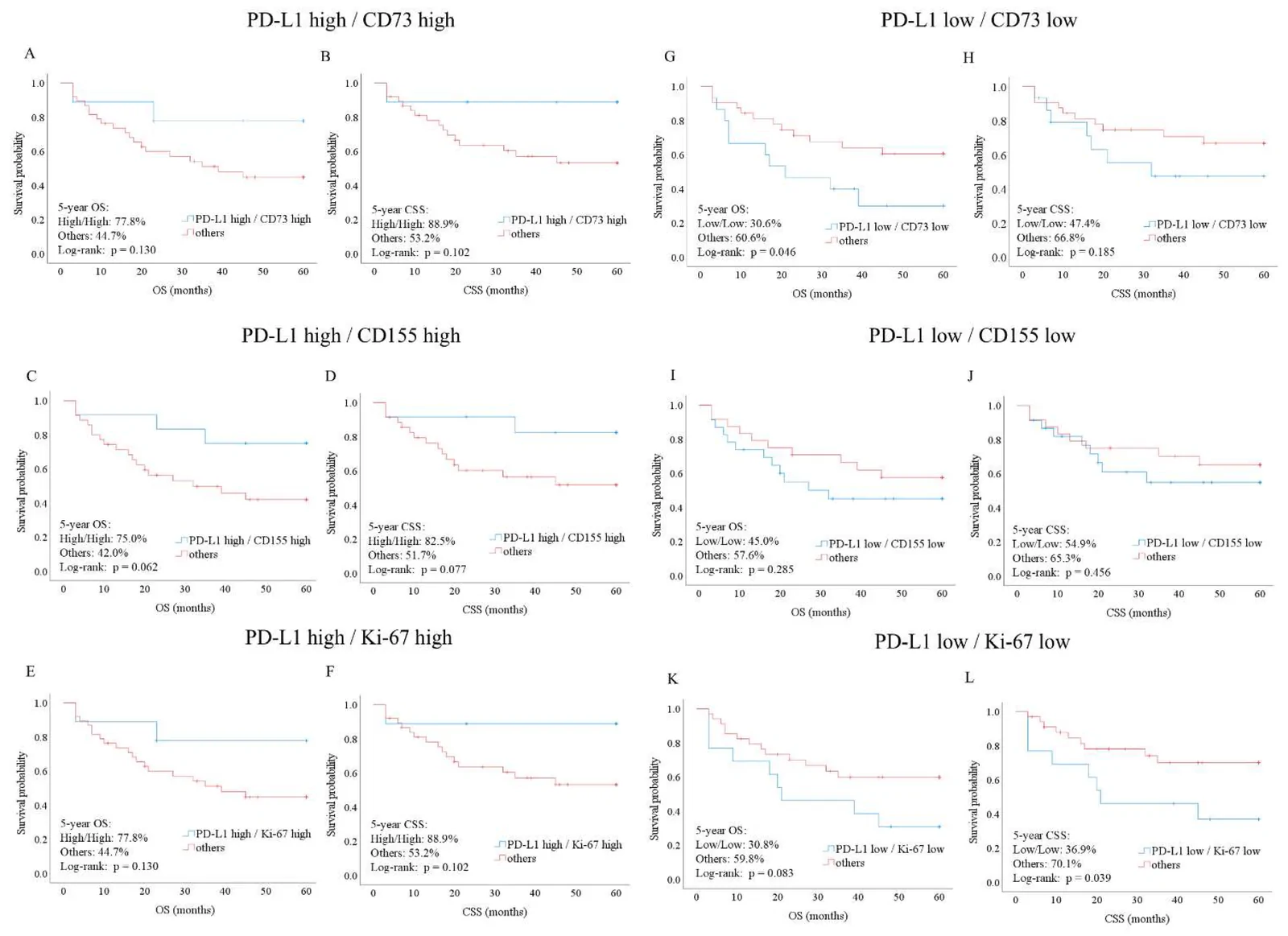
Kaplan–Meier curves for overall survival and cancer-specific survival according to concurrent high or low expression of PD-L1 and one additional biomarker. *p* values were calculated using the log-rank test. Panels (**A**–**F**) compare cases with concurrent high expression of both markers with all other cases, and panels (**G**–**L**) compare cases with concurrent low expression of both markers with all other cases. (**A**,**G**) PD-L1 and CD73 for overall survival. (**B**,**H**) PD-L1 and CD73 for cancer-specific survival. (**C**,**I**) PD-L1 and CD155 for overall survival. (**D**,**J**) PD-L1 and CD155 for cancer-specific survival. (**E**,**K**) PD-L1 and Ki-67 for overall survival. (**F**,**L**) PD-L1 and Ki-67 for cancer-specific survival.

High PD-L1 paired with high CD73, CD155, or Ki-67 was not associated with significantly better OS/CSS (Figure 4).

In contrast, low PD-L1 and low CD73 had significantly worse OS than the other PD-L1×CD73 groupings (5-year OS = 30.6 vs. 60.6%; *p* = 0.046) (Figure 4G). Low PD-L1 and low Ki-67 were associated with significantly worse CSS than other PD-L1 × Ki-67 constellations (5-year CSS = 36.9 vs. 70.1%; *p* = 0.039) (Figure 4L).

### 3.7. Independent Prognostic Factors

Cox analyses (Table 2) showed no statistically significant associations with OS or CSS for any of the variables examined.

## 4. Discussion

### 4.1. Principal Findings

We found high expression of PD-L1, CD73, CD155, and Ki-67 in resected PPCs. In the primary endpoint analysis, no individual marker showed a statistically significant association with OS or CSS. In contrast, secondary endpoint analysis of combinations of the studied biomarkers identified poor-risk subsets that were not detected when these markers were assessed individually. Low PD-L1 with low CD73 was associated with significantly worse OS, and low PD-L1 with low Ki-67 was associated with significantly worse CSS. These findings suggest that evaluating more than one marker may be more useful than relying on a single marker.

### 4.2. Comparison with Previous Studies

Our findings concerning PD-L1 are in line with previous reports on PPC. Imanishi et al. reported frequent PD-L1 positivity in pleomorphic carcinoma of the lung and suggested that higher PD-L1 expression may be associated with a favorable prognosis after resection [11]. Chen and Mellman reported suggestive evidence that tumors with an inflamed tumor microenvironment are more likely to mount effective antitumor immune responses [20]. This is one possible explanation for the tendency for higher PD-L1 expression to be associated with better outcomes in our series. However, we could not investigate the underlying mechanism in the present study. The present study is important because it provides some evidence for associations between prognosis and biomarkers other than PD-L1. To our knowledge, only a few PPC-specific studies have systematically used immunohistochemistry to evaluate CD73. Furthermore, major reviews of PSC/PPC have not summarized clear clinicopathologic data on associations between CD73 and these histologic tumor types [1,26,27]. In contrast, in NSCLC, Inoue et al. reported an association between a combination of high expression of both CD73 and A2A receptor and a poor prognosis, supporting the contention that the adenosine pathway has clinical relevance in lung cancer [12]. These findings have also led to the use of adenosine-related strategies in combination with PD-1/PD-L1 blockade in the clinic [13]. However, in our cohort the group with low PD-L1 and low CD73 had the poorest OS, not the group with high CD73 only. This finding suggests that CD73 may be more useful when combined with PD-L1 than when used as a sole marker. One possible explanation for these findings is that tumors with concurrent low expression of both markers may reflect a relatively “cold” tumor microenvironment. Recent studies have categorized tumors as “hot” or “cold” according to the degree of immune cell infiltration and immune activation within the tumor microenvironment [28]. In general, cold tumors tend to show limited immune cell infiltration and reduced antitumor immune responses, which may contribute to poorer clinical outcomes [29]. In the present study, the PD-L1/CD73 low/low subgroup may have represented a less immune-active tumor microenvironment, potentially resulting in reduced immune surveillance and poorer overall survival. Although tumor-infiltrating lymphocytes were not directly evaluated in this study, the hot/cold tumor concept may provide one possible interpretation of our findings. Another marker, CD155, also deserves attention. As with CD73, we found very few PPC-specific published studies in which immunohistochemistry was used to systematically assess CD155 expression. Furthermore, available reviews of the management of PSC/PPC do not clearly summarize the role of CD155 in these cancers [26,27]. Despite this, the biologic relevance of CD155 is now well established. Yeo et al. have described the role of the TIGIT/CD226 axis in antitumor immunity [14] and Zhang et al. have reviewed recent progress in targeting TIGIT for cancer immunotherapy [15]. In addition, Freed-Pastor et al. have shown that the CD155/TIGIT axis can promote and maintain immune escape in vivo [30]. These findings support the importance of CD155 as an immune-regulatory ligand. In our cohort, associations between CD155 as a single prognostic marker and prognosis were not statistically significant. This negative result should be interpreted with caution because of the small sample size and the rarity of PPC. Rather than indicating that CD155 is unimportant, we believe that our data suggest that, in this cohort, PD-L1-based combinations were more clearly associated with prognosis than CD155 was alone. We recommend including CD155 in staining frameworks because the findings would establish a basis for future investigation into the role of combinations of biomarkers in PPC. Importantly, we included Ki-67 in the present study because it is widely used as an indicator of proliferative activity. Furthermore, many studies of NSCLC have shown that higher Ki-67 expression is associated with more aggressive behavior and a worse prognosis [31,32]. Regarding PPC, Pelosi et al. reported that a high Ki-67 labeling index (>35%) is associated with a worse prognosis [33], but its role in this rare histological type remains less well characterized than in conventional NSCLC. In our study, Ki-67 alone was not significantly associated with OS or CSS. However, when we combined it with PD-L1, a significant association was observed. Specifically, low PD-L1 with low Ki-67 was associated with a significantly worse CSS. This finding is important because it indicates that low proliferative activity does not necessarily imply favorable prognosis. A tumor with low Ki-67 and an unfavorable immune microenvironment may still behave aggressively. Mariathasan et al. showed that stromal transforming growth factor-beta signaling can drive T-cell exclusion and limit the effect of PD-L1 blockade [21]. We did not evaluate stromal factors in the present study, despite Mariathasan et al.’s findings supporting the general contention that the tumor microenvironment can influence prognosis independently of proliferation itself. From a practical point of view, Ki-67 is an attractive marker to investigate because it is routinely available in pathology laboratories and can easily be assessed in the same operative specimen as other markers. Thus, the PD-L1 and Ki-67 combination may provide a simple and practical way to refine risk stratification in patients with PPC. Another important point is that our study is consistent with the broader trend toward assessment of combinations of biomarkers in oncology. Shirasawa et al. have shown that the immune-related tumor microenvironment influences the efficacy of anti-PD-1/PD-L1 therapy in NSCLC, highlighting the value of combining tumor- and immune-related approaches [17]. Cristescu et al. have demonstrated that genomic biomarkers such as tumor mutational burden and T-cell-inflamed gene expression profile can complement PD-L1-related assessment in predicting response to PD-1 blockade [18]. Lu et al. demonstrated that different biomarker modalities contribute complementary information for predicting response to PD-1/PD-L1 blockade, supporting the importance of integrated biomarker assessment [19]. The findings of other studies in which epithelial PD-L1 scoring is combined with immune cell features have supported the above [22]. Reviews by Nishino et al. and Doroshow et al. have further emphasized the limitations of single biomarker approaches and the complexity of immunotherapy-related biology, supporting the need for integrated biomarker assessment [23,24]. Our findings in PPC are generally consistent with those of other studies in other tumor types. In particular, the combinations of PD-L1 with CD73 and PD-L1 with Ki-67 identified prognostic subgroups that were not identified by PD-L1 alone. The present findings should also be considered in the context of existing published reports on PSC/PPC. PSC, including PPC, is immunologically distinctive and clinically challenging. Recent reviews have highlighted frequent immune checkpoint expression and the emerging role of immunotherapy in these tumors, while also emphasizing the lack of prospective studies of biomarkers in this field [1,26,27]. In light of this, our study is valuable in two respects. First, we systematically assessed CD73, CD155, and Ki-67 together with PD-L1 in PPC. Second, we evaluated associations of prespecified pairwise combinations with survival outcomes. To our knowledge, few published studies have analyzed this type of data in PPC. In our study cohort, the combinations of PD-L1 with CD73 and PD-L1 with Ki-67 had clearer prognostic impacts than did a PD-L1/CD155 combination. The combination of PD-L1 with CD73 may be of particular interest because this grouping identified a prognostic subgroup that was not identified by assessing PD-L1 alone; thus, it may better reflect differences in the tumor microenvironment. PD-L1 with Ki-67 may also be useful because it combines immune- and proliferation-related markers. However, given the small sample size, the present findings should be interpreted cautiously and not taken to indicate that one combination is definitively superior to another.

### 4.3. Clinical Implications and Future Perspectives

Our findings have several clinical implications. Prognostic stratification of patients with PPC may be improved by biomarker assessment beyond PD-L1 alone. First, the combinations of PD-L1 with CD73 and PD-L1 with Ki-67 are likely to help identify patients with worse prognoses after resection. Second, the combination of PD-L1 and Ki-67 is attractive from a practical perspective because both of these markers are already used in routine pathology practice. Furthermore, a combination of PD-L1 and CD73 may provide additional information by identifying a prognostic subgroup that was not revealed by PD-L1 alone. Although we found no statistically significant evidence that CD155 has prognostic value in the present cohort, its current clinical relevance as a therapeutic target in lung cancer warrants further exploration of combinations of biomarkers in future studies. While the present study evaluated tissue expression by immunohistochemistry, future studies may investigate whether these biomarkers, or their soluble forms, could be assessed using liquid biopsy approaches to provide less invasive, real-time prognostic monitoring. Importantly, these potential roles should be regarded as exploratory and would require validation in larger cohorts.

### 4.4. Limitations

This study had some limitations. It was a retrospective single institution study, and the cohort was small because PPC is rare. The cut-off values were determined by ROC analysis using the same retrospective cohort and were not externally validated. Therefore, these thresholds should be considered exploratory and hypothesis-generating rather than definitive and require validation in independent cohorts. Furthermore, the long period of accrual spanning over two decades would inevitably have resulted in treatment being heterogeneous, introducing potential confounding factors related to treatment-era effects. Because multiple comparisons were performed without formal adjustment, the possibility of false-positive findings cannot be excluded. In addition, we did not assess spatial immune features or functional immune readouts. These limitations mean that our findings should be interpreted cautiously and validated in larger multi-institutional cohorts.

## 5. Conclusions

Single selected markers (PD-L1, CD73, CD155, and Ki-67) examined in resected PPC showed no statistically significant association with prognosis. In contrast, combinations of these markers were associated with poorer outcomes in the low PD-L1 with low CD73 (OS) and low PD-L1 with low Ki-67 (CSS), subgroups that were not revealed by single markers. These findings are preliminary and provide a basis for further investigation of biomarker combinations for prognostic assessment in this rare lung cancer. Because of the limited sample size, they should be regarded as exploratory and require validation in larger independent cohorts.

## Figures and Tables

**Table 1:** Relevant patient and tumor characteristics. Patient demographics and baseline clinical characteristics.

Parameter	*n* = 47	%/Range
Age, years, median	72	35–91
Gender (male)	38	80.9
ECOG PS		
0	29	61.7
1	12	25.5
2	4	8.5
3	2	4.3
Type of resection		
R0	44	93.6
R1 (chest wall)	3	6.4
Pathologic TNM (8th edition)		
I	12	25.5
II	17	36.2
III	18	38.3
Pathologic T stage		
T1	10	21.3
T2	12	25.5
T3–4	25	53.2
Pathologic N stage		
N0	32	68.1
N1	9	19.2
N2	5	10.6
N3	1	2.1
Histologic types		
PPC	15	31.9
PPC + adenocarcinoma	29	61.7
PPC + squamous cell carcinoma	3	6.4
Perioperative therapy		
None	24	51.1
Neoadjuvant therapy	5	10.6
Adjuvant therapy	18	38.3
Positive expression of molecules		
PD-L1	33	70.2
CD73	42	89.4
CD155	43	91.5
Ki-67	39	83.0

Abbreviation: PPC, pulmonary pleomorphic carcinoma.

**Table 2:** Cox proportional hazards analysis of prognostic factors for overall survival and cancer-specific survival in patients with pulmonary pleomorphic carcinoma.

	Prognostic Factors on OS in PPC				Prognostic Factors on CSS in PPC			
	Cox Univariate		Cox Multivariate		Cox Univariate		Cox Multivariate	
Parameter	HR (95%CI)	*p* Value	HR (95%CI)	*p* Value	HR (95%CI)	*p* Value	HR (95%CI)	*p* Value
Age (≤65 vs. ≥66 years)	1.50 (0.59–3.85)	0.395			1.02 (0.38–2.77)	0.965		
Gender (female vs. male)	0.85 (0.29–2.51)	0.769			0.84 (0.24–2.91)	0.776		
ECOG PS (0 vs. 1–3)	2.04 (0.88–4.72)	0.095	1.96 (0.84–4.53)	0.118	2.27 (0.87–5.88)	0.093	2.34 (0.89–6.15)	0.086
Type of resection (R0 vs. R1)	1.86 (0.43–8.03)	0.396			2.51 (0.57–11.1)	0.225		
Histologic types (PPC vs. PPC + ad/sq)	0.88 (0.37–2.10)	0.776			0.91 (0.34–2.45)	0.846		
pathologic Stage (I–II vs. III)	1.60 (0.69–3.72)	0.279			2.12 (0.81–5.51)	0.117		
PD-L1 (<15% vs. 15%≤)	0.41 (0.19–1.22)	0.108			0.40 (0.12–1.40)	0.153		
CD73 (<25% vs. 25%≤)	0.54 (0.23–1.26)	0.153			0.58 (0.22–1.50)	0.262		
CD155 (<50% vs. 50%≤)	0.71 (0.30–1.65)	0.420			0.61 (0.23–1.62)	0.322		
Ki-67 (<5% vs. 5%≤)	0.60 (0.26–1.39)	0.236			0.43 (0.16–1.12)	0.084	1.25 (0.35–4.44)	0.731
Neoadjuvant therapy (none vs. performed)	2.08 (0.70–6.15)	0.186			1.96 (0.56–6.81)	0.292		
Adjuvant therapy (none vs. performed)	0.37 (0.14–1.01)	0.052	0.384 (0.14–1.05)	0.061	0.53 (0.19–1.52)	0.240		

Abbreviations: ad, adenocarcinoma; CSS, cancer-specific survival; OS, overall survival; PPC, pulmonary pleomorphic carcinoma; sq, squamous cell carcinoma.

## Data Availability

The datasets used and/or analyzed during the present study are available from the corresponding author on reasonable request.
